# Complete Genome Sequence of Methicillin-Resistant *Staphylococcus schleiferi* Strain TSCC54 of Canine Origin

**DOI:** 10.1128/genomeA.01268-15

**Published:** 2015-10-29

**Authors:** Takashi Sasaki, Sae Tsubakishita, Kyoko Kuwahara-Arai, Miki Matsuo, Yu Jie Lu, Yoshikazu Tanaka, Keiichi Hiramatsu

**Affiliations:** a Department of Microbiology, Faculty of Medicine, Juntendo University, Tokyo, Japan; b Department of Veterinary Science, School of Veterinary Medicine, Rakuno Gakuen University, Hokkaido, Japan; c Department of Veterinary Hygiene, Veterinary School, Nippon Veterinary and Life Science University, Tokyo, Japan

## Abstract

We report a complete genome sequence of the methicillin-resistant *Staphylococcus schleiferi* strain TSCC54, isolated from the skin of a dog in Tokyo, Japan.

## GENOME ANNOUNCEMENT

*Staphylococcus schleiferi*, which consists of two subspecies, *schleiferi* and *coagulans*, distinguished by coagulase activity, was taxonomically described as a novel species recovered from humans and dogs, respectively (1, 2). However, *S. schleiferi* is not common in human medicine, and once the infection occurs in humans, it produces serious symptoms (3–7). On the other hand, *S. schleiferi* is the secondary dominant species after *S. pseudintermedius* in canine hosts, and it causes various disease states such as pyoderma, otitis, and urinary tract infections. The isolation of methicillin-resistant *S. schleiferi* (MRSS) strains is also now increasing in dog infections (8).

The genome of the MRSS strain TSCC54 was isolated from a dog skin in Japan and was sequenced using a Roche 454 GS FLX Titanium sequencer (8-kbp mate-pair library; average read length, 168 bp; 350,963 reads) and an Illumina HiSeq 2000 platform (100-mer single-end reads; 6,808,914 reads). *De novo* genome hybrid assembly using the GS *de novo* Assembler version 2.6 produced one scaffold composed of 27 contigs and gaps. Sequences within the 27 gaps were determined by an qjABI 3730 sequencer. Gene annotation was performed on the Rapid Annotations using Subsystems Technology (RAST) server (9) and the KEGG Automatic Annotation Server (KAAS) (10).

The genome of strain TSCC54 had a size of 2,528,077 bp and exhibited high G+C content (35.9%) in contrast to *S. aureus*, many of which have 32.8 to 33.0% (11). This genome possesses 2,364 open reading frames, 5 ribosomal operons, and 58 tRNAs. The structures of staphylococcal cassette chromosome *mec* (SCC*mec*) was similar to that of type V SCC*mec* found in the livestock-associated methicillin-resistant *S. aureus* (LA-MRSA) strain 08BA02176 having a genotype of ST398 (12).

This deposit of an *S. schleiferi* genome in GenBank will contribute to the studies of various fields, such as genomics, ecology, epidemiology, and infectious disease.

### Nucleotide sequence accession number. 

The complete genome sequence of *S. schleiferi* strain TSCC54 has been deposited in GenBank under the accession number AP014944.
